# How Neighborhood Cohesion and Disorder Shape Late-Life Cognition: The Mediating Role of Psychological Resilience

**DOI:** 10.1093/geroni/igaf122.2722

**Published:** 2025-12-31

**Authors:** Guoping Jin, Xiaoyi Zeng, Qingqing Yin, Fengyan Tang

**Affiliations:** University of Pittsburgh, Pittsburgh, Pennsylvania, United States; The University of Texas at Austin, Austin, Texas, United States; University of Pittsburgh, Pittsburgh, Pennsylvania, United States; University of Pittsburgh, Pittsburgh, Pennsylvania, United States

## Abstract

Although prior research has linked neighborhood environments to cognitive health in later life, the mechanisms under these associations are less well understood. Drawing on the theoretical concept of resilience, this study examined whether psychological resilience mediated the effects of perceived neighborhood cohesion and disorder on cognitive functioning among older adults. Using the Health and Retirement Study 2010-2018 Leave Behind Questionnaire and RAND Data File, this study included 4,708 respondents aged 65 and older at baseline. Cognitive functioning was assessed using a modified 35-point Telephone Interview Cognitive Screen, while psychological resilience was measured with a simplified resilience scale based on prior research. Using path analyses, this study examined the potential direct and indirect effects of Time 1 neighborhood environment (predictor) and Time 2 psychological resilience (mediator) on Time 3 cognitive functioning (outcome), adjusting for sociodemographic and health-related covariates. The results indicated that greater neighborhood cohesion was significantly associated with higher levels of cognitive functioning (β = 0.03, p < .001), with psychological resilience partially mediating this effect (indirect effect = 0.01, p < .001). In contrast, neighborhood disorder was not significantly associated with cognitive functioning (β = 0.1, p > .05). These findings advance our understanding of the mechanisms through which neighborhood environments influence cognitive health in later life. Enhancing resilience may help leverage the cognitive benefits of neighborhood cohesion. Other implications for practice and future research are discussed.

